# Supplementary material for: Causal Relationship between Aging and Anorexia Nervosa: A White-Matter-Microstructure-Mediated Mendelian Randomization Analysis
Source: Biomedicines. 2024 Aug 16;12(8):1874. doi: 10.3390/biomedicines12081874 (PMC11351342; doi:10.3390/biomedicines12081874)
Supplement: Supplementary file 1 [file biomedicines-12-01874-s001.zip › Supplementary Figure/Figure S8/Leaveoneout plot of MR analysis for TL to ubm-b-1550.pdf]

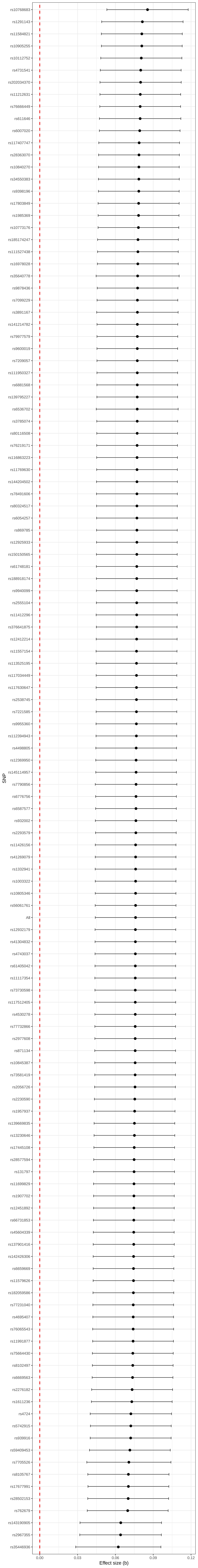

rs10768683
rs1291143
rs11584821
rs10905255
rs10112752
rs4731541
rs202034370
rs11212631
rs76666449
rs611646
rs6007020
rs117407747
rs28363070
rs10840270
rs34550383
rs9398196
rs17803849
rs1985369
rs10773176
rs185174247
rs111527438
rs16978028
rs35640778
rs9878436
rs7099229
rs3891167
rs141214782
rs79977579
rs9600019
rs7209057
rs111950327
rs6881568
rs139795227
rs6536702
rs3785074
rs80116508
rs76219171
rs116863223
rs11769630
rs144204502
rs78491606
rs80324517
rs6054257
rs869785
rs12925933
rs150150565
rs61748181
rs188918174
rs9940099
rs2555104
rs11412296
rs376641875
rs12412214
rs11557154
rs113525195
rs117034449
rs117630647
rs2538745
rs7221585
rs9955360
rs112394943
rs4498805
rs12369950
rs145114957
rs7790856
rs6776756
rs6587577
rs932002
rs2293579
rs11426156
rs41269079
rs1332941
rs1003322
rs10805346
rs56061761
All
rs12932179
rs41304832
rs4743037
rs61405042
rs11117354
rs73730598
rs117512405
rs4530278
rs77732866
rs2977608
rs871134
rs10845387
rs73581419
rs2056726
rs2230590
rs1957937
rs139669835
rs13230646
rs17445108
rs28577594
rs131797
rs11699829
rs1907702
rs12451892
rs66731853
rs45604339
rs137901416
rs142426306
rs6659669
rs11579626
rs182059586
rs77231040
rs4695407
rs76065543
rs11991877
rs75664430
rs8102497
rs6669563
rs2276182
rs1611236
rs4724
rs5742915
rs939916
rs59409453
rs7705526
rs8105767
rs17677991
rs28502153
rs762679
rs143190905
rs2967355
rs35446936
SNP
0.00
0.03
0.06
0.09
0.12
Effect size (b)
